# Comparison of time-segmented goal-directed teaching vs. traditional teaching for PICC among specialist nurse training: a randomized controlled trial

**DOI:** 10.3389/fmed.2026.1798774

**Published:** 2026-04-13

**Authors:** Chang Xie, Jie Zhang, Ge Long, Xi He, Xing Liu, Mi Yang

**Affiliations:** 1 Health Management Medical Center, The Third Xiangya Hospital, Central South University, Changsha, Hunan, China; 2 Department of Gastrointestinal Surgery, The Third Xiangya Hospital, Central South University, Changsha, Hunan, China; 3 Department of Anesthesiology, The Third Xiangya Hospital, Central South University, Changsha, Hunan, China

**Keywords:** peripherally inserted central catheter, randomized controlled trial, specialist nurse training, time-segmented goal-directed teaching, traditional teaching

## Abstract

**Introduction:**

This randomized controlled trial compared the efficacy of Time-Segmented Goal-Directed Teaching (TSGDT) vs. Traditional Teaching (TT) in enhancing Peripherally Inserted Central Catheter (PICC) skills among specialist nurse trainees.

**Methods:**

A total of 104 specialist nurse trainees were randomized to TSGDT (*n* = 52) or TT (*n* = 52). TSGDT group segmented PICC operation into three temporally defined phases (pre-operative preparation, operational phase, and post procedure management & holistic care), each with explicit performance criteria and time-bound practice. TT delivered sequential didactic instruction of the entire procedure. Both groups received 4 hours of training. Primary outcome was Objective Structured Clinical Examination (OSCE) performance of PICC. Secondary outcomes included knowledge assessment, willingness, perceived confidence, and training satisfaction. Outcomes were measured immediately post-intervention and at 4-week follow-up.

**Results:**

Compared with the TT group, participants in the TSGDT group achieved significantly higher overall OSCE scores immediately after training (*p* < 0.0001). This advantage was evident across all key domains of pre-procedure preparation (*p* = 0.015), operational phase (*p* = 0.0017), and post procedure management/holistic care (*p* = 0.010). Additionally, participants in TSGDT group completed the OSCE scenario in significantly less time (*p* = 0.018). At the 4-week follow-up, the TSGDT group maintained significantly higher OSCE performance than the TT group (*p* < 0.0001). Notably, the magnitude of skill decay from immediate post-test to follow-up was significantly smaller in the TSGDT group (*p* < 0.0001), indicating enhanced skill retention and consolidation. However, no significant between-group difference was observed in theoretical knowledge test scores (*p* = 0.238). Regarding subjective outcomes, TSGDT participants reported significantly greater confidence in providing PICC care (*p* = 0.026), willingness to perform PICC under supervision (*p* = 0.030), perceived mastery (*p* = 0.038), and overall satisfaction with the training program (*p* = 0.027) compared to their TT counterparts.

**Conclusion:**

The Time-Segmented Goal-Directed Teaching method is a highly effective instructional strategy that significantly enhances PICC procedural competence, procedural confidence, and learning satisfaction among specialist nurse trainees, with superior skill retention at 4 weeks, which offering a superior alternative to traditional teaching for clinical skill acquisition.

## 1 Introduction

Peripherally Inserted Central Catheter (PICC) represents a high-stakes, evidence-based nursing competency critical for safe long-term vascular access in oncology, critical care, and nutritional support (1–3). Mastery of this complex psychomotor skill is indispensable for specialist nurses, who bear direct responsibility for procedural safety and patient outcomes (4). Yet, current training paradigms consistently fail to bridge the critical gap between theoretical knowledge and reliable clinical performance. A significant proportion of specialist nurse trainees enter practice with suboptimal PICC proficiency, evidenced by high rates of procedural anxiety, variable competence, and elevated risk of complications (5, 6). Crucially, this deficiency is not merely a training shortfall, but also posing a systemic threat to patient safety (7). Graduates with fragmented skills and low self-efficacy are ill-prepared to perform independently, mentor peers, or uphold clinical standards, thereby contributing to preventable adverse events. Recent data underscore this urgency: inadequate initial PICC training is associated with a 42% increase in early procedural complications and a 68% longer clinical learning curve (8), highlighting an unmet imperative for transformative pedagogy.

The prevailing traditional teaching (TT) approach relies on sequential didactic lectures, instructor demonstrations, and supervised practice (9). This instructor-centered model prioritizes knowledge delivery over skill consolidation, often failing to address individual learning trajectories, cognitive overload during complex procedures, or the need for structured feedback (10). Consequently, trainees experience fragmented skill integration, poor long-term retention, with performance declining by 25–30% within four weeks, and persistent confidence deficits, all of which undermine the transition to independent practice.

To resolve this crisis, we developed Time-Segmented Goal-Directed Teaching (TSGDT), an evidence-based intervention grounded in mastery learning and cognitive load theory (11, 12). TSGDT deconstructs PICC insertion into discrete, temporally sequenced phases (pre-operative preparation, operational phase, and post procedure management & holistic care), each with clearly defined, measurable performance criteria and limited time. Trainees engage in repetitive, criterion-referenced practice within each segment, receiving immediate corrective feedback until mastery is achieved before progression. This approach delivers three synergistic outcomes:

- **Reduced cognitive load** through phase-specific focus, enabling deeper skill automation;
- **Enhanced skill retention** via deliberate practice, minimizing knowledge decay;
- **Elevated self-efficacy** through incremental mastery, directly addressing procedural anxiety.

Critically, TSGDT shifts training from passive knowledge transfer to active competency construction—a paradigm essential for preparing specialist nurses to deliver safe, confident care from their first independent procedure.

This randomized controlled trial (RCT) rigorously evaluates TSGDT against TT in a high-stakes clinical context, directly addressing the urgent need for a scalable, evidence-based solution to a pervasive training deficit. By demonstrating superior immediate skill acquisition, sustained retention, and enhanced learner confidence, this study provides actionable evidence to transform PICC education—and by extension, the safety and efficacy of vascular access care worldwide.

## 2 Methods

### 2.1 Study design

**Figure 1:**
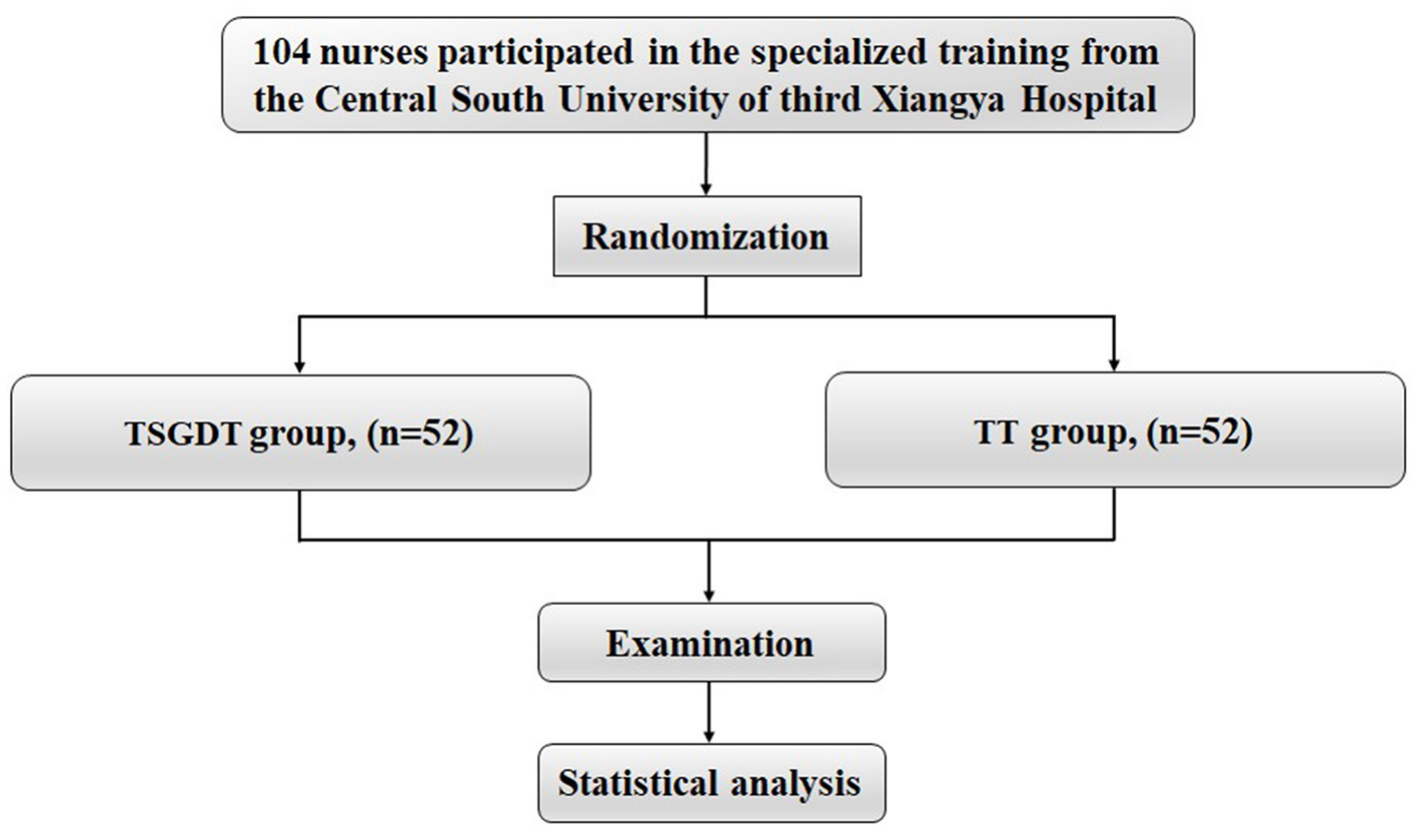
Enrollment, randomization, and protocol of participants.

This study was a prospective, parallel-group randomized controlled trial. We implemented the Time-Segmented Goal-Directed Teaching (TSGDT) method as an educational intervention for specialist nurses enrolled in a standardized PICC training program at a university-affiliated simulation center. All participating nurses were randomly assigned (1:1) to either the Traditional Teaching (TT) group or the TSGDT group, ensuring no overlap between groups (Figure 1). The TT group received the standard, instructor-led PICC curriculum, while the TSGDT group participated in the structured TSGDT program. Each group was supervised by an experienced nurse educator with over five years of experience in PICC training and simulation education. Both faculty members had completed a standardized “Train-the-Trainer” workshop to ensure methodological fidelity. They were provided with detailed, group-specific lesson plans and protocols to ensure standardization. Their role was to facilitate learning according to the assigned methodology, provide structured feedback (TSGDT group) or general guidance (TT group), and ensure a safe learning environment.

The total intervention duration for both groups was 4 h of supervised training. The training was conducted in the clinical skills simulation center, which was equipped with vascular access phantoms, and full PICC supplies. Each instructor was responsible for a cohort of 18 trainees, resulting in a facilitator-to-student ratio of 1:18. The study was conducted in accordance with the 2010 Consolidated Standards of Reporting Trials (CONSORT) reporting guidelines. Participation was voluntary, and no incentives were offered. Program attendance, adherence to the teaching protocol, and time-on-task were monitored by a trained research assistant who was not involved in instruction or assessment.

### 2.2 Participants

All participants were registered nurses enrolled in a “Specialist Nurse for Intravenous Therapy” training program. They had a minimum of two years of clinical experience. According to the national core competency framework for specialist nurses, proficient and independent performance of PICC is a mandatory clinical skill.

Recruitment was open to two consecutive cohorts of the training program from April 12st, 2024, to May 30st, 2025. To ensure baseline comparability, we excluded nurses who had previously learning experience in PICC or had received formal training in simulation-based mastery learning methodologies. All eligible nurses who volunteered provided written informed consent.

Randomization and Allocation Concealment: Participants were randomly assigned in a 1:1 ratio to either the TSGDT group or the TT group. The random allocation sequence was generated by an independent staff not involved in the recruitment, intervention, or assessment processes using a computer-generated random number table (SAS software, Version 9.4, SAS Institute, Cary, NC). To ensure allocation concealment, the sequence was placed in sequentially numbered, opaque, sealed envelopes, which were stored by an independent research coordinator. After a participant was confirmed eligible and baseline assessments were completed, the recruiter contacted the coordinator, who then opened the envelope to reveal the group assignment. Given the nature of the educational intervention, it was not possible to blind the participants or the instructors to the group allocation. However, outcome assessors and data analysts were blinded to group assignment. All outcome assessments, including the Objective Structured Clinical Examination (OSCE) and theoretical knowledge tests, were evaluated by trained clinical faculty who were not involved in the teaching process and were unaware of the participants' group allocation.

### 2.3 Study protocol

Both the Traditional Teaching (TT) and Time-Segmented Goal-Directed Teaching (TSGDT) programs were designed to be of equal total duration (4 h) and were delivered to groups of 10 to12 participants to maintain consistency in instructor-to-learner ratio. Prior to the conclusion of the 4-h course, a different instructor, who was blinded to group allocation, conducted a summative evaluation and assigned a score to each participant based on their demonstrated skill level.

#### 2.3.1 Traditional teaching (TT) group

The TT group received instruction based on a conventional, procedure-focused apprenticeship model. The 4-h session was structured as follows:

- **Didactic lecture (0.5 h):** The session commenced with a comprehensive, instructor-led lecture covering the theoretical foundations of PICC, including indications, contraindications, relevant anatomy, ultrasound physics, procedural steps, potential complications, and post-insertion care protocols.
- **Procedural demonstration (0.5 h):** Following the lecture, the instructor provided a step-by-step, uninterrupted demonstration of the entire PICC insertion procedure on a task trainer, explaining each action according to clinical standards.
- **Supervised practice with summative feedback (3 h):** Participants then engaged in hands-on practice on the model under direct instructor supervision. The remaining time (3 h) was dedicated to this practice. Instructors observed performance and provided summative, end-of-session feedback after each practice attempt, focusing on the overall execution of the procedure.

#### 2.3.2 Time-segmented goal-directed teaching (TSGDT) group

The TSGDT group received the same foundational theoretical content but with a restructured practical component. The 4-h session was segmented into distinct, time-bound phases, each with explicit, measurable performance goals that had to be met before progression.

- **Foundation (0.5 h):** The session began with a didactic lecture covering the same essential theoretical content as the TT group to ensure a consistent knowledge baseline.
- **Segmented instruction and deliberate practice (3 h):** The instructor then deconstructed the PICC procedure into three sequential, goal-oriented stages. For each stage, the instructor provided a focused demonstration, after which participants practiced that specific segment on the model to achieve a predefined time-based mastery standard.

- **Phase 1: pre-operative preparation (0.5 h):** The instructor demonstrated and explained the comprehensive preparations required for the operator, patient, and supplies. Participants were then tasked with practicing and mastering this segment, with the goal of completing all preparatory steps accurately within **1 min**.
- **Phase 2: procedural execution (1 h):** Detailed guidance and demonstration were provided for the core operational steps, including patient positioning, disinfection, draping, local anesthesia, ultrasound-guided puncture, and catheterization. Participants practiced these steps, aiming to complete the procedural execution accurately within **6 min**.
- **Phase 3: post-procedure management & holistic care (0.5 h):** Instruction focused on post-procedure protocols, catheter securement, documentation, and patient communication (holistic care). Participants practiced these concluding tasks with the goal of accurate completion within **1 min**.

- **Integrated practice (0.5 h):** After mastering each individual segment, participants dedicated the final hour to practicing the entire PICC procedure from start to finish, integrating all three phases into a seamless workflow. The instructor's role during this phase was to facilitate reflection, provide formative feedback, and ensure consolidation of learning.

The time allocation and mastery standards for each segment were derived from expert consensus and validated through a pilot study with 10 nurses (not part of the main trial). The instructor's role was to manage the progression, ensure goal attainment before advancing, and facilitate reflection. Mastery for each segment was defined by a dual criterion: (1) successful completion of all essential steps within the prescribed time limit, and (2) error-free performance of critical actions. A trainee could only advance to the next phase upon successfully demonstrating mastery of the current one, ensuring the consolidation of psychomotor skills and cognitive knowledge before encountering more complex tasks. Before the end of the course, another instructor who was blinded to the grouping conducted an evaluation, assigning a score to each participant based on their demonstrated skill level.

### 2.4 Outcome measures

Before the training, participant demographics and baseline characteristics were recorded, including age, gender, years of nursing experience, previous experience with intravenous cannulation, and scores on a pre-test of theoretical PICC knowledge. The primary outcome was Objective Structured Clinical Examination (OSCE) for PICC immediately and at 4 weeks after training. Secondary outcomes were estimated by knowledge test, subjective effects and satisfaction. The OSCE for PICC, scored by blinded evaluators, included stations on patient assessment, aseptic technique, procedural execution, and patient communication (see **Appendix 1**). Subjective effects and satisfaction were evaluated by questionnaire survey after training (see **Appendix 2**). A written knowledge test is also conducted after the training is completed.

The assessment tools were adapted from validated instruments. The OSCE for PICC was developed based on national guidelines and showed excellent inter-rater reliability (Cohen's κ = 0.89) during piloting. The subjective effects and satisfaction survey demonstrated good internal consistency (Cronbach's α = 0.87 and 0.82, respectively).

### 2.5 Ethics

The trial was registered and approved by the Institutional Review Board of the Third Xiangya Hospital of Central South University (Date: 03/14/2024, Decision No: R24043). The studies were conducted in accordance with the local legislation and institutional requirements. The participants provided their written informed consent to participate in this study.

### 2.6 Statistical analysis

Based on a pilot study, the expected mean difference in the primary OSCE score (100-point scale) between the TT and TSGDT groups was 7.3 points, with a pooled standard deviation of 11.0. For a two-tailed test with Effect size *d* = 0.62, α = 0.05 and power (1-β) = 0.80, and assuming a 10% dropout rate, a minimum of 52 participants per group (total *N* = 104) was required.

Analyses were performed using IBM SPSS Statistics (version 29.0). Continuous variables were tested for normality. Between-group comparisons for primary and secondary outcomes at post-test were made using independent samples *t*-tests or Mann-Whitney U tests. Within-group changes across time (pre-test, post-test, 1-month follow-up for OSCE) were analyzed using repeated-measures ANOVA or Friedman tests. Categorical data were compared using the χ^2^ test. A p-value < 0.05 was considered statistically significant. All analyses followed the intention-to-treat principle.

## 3 Results

### 3.1 Demographic characteristics of participants

A total of 104 specialist nurse trainees completed the study protocol and all post-intervention assessments. No significant differences existed between the Traditional Teaching (TT) group and the Time-Segmented Goal-Directed Teaching (TSGDT) group in age, gender, education level, or clinical nursing experience (all *p* > 0.05, showed in Table 1). Baseline PICC experience revealed that 65.4% (*n* = 68) of participants had no prior exposure to PICC procedures, while 34.6% (*n* = 26) had observed but not performed PICC. None had received formal simulation-based mastery learning training. No protocol deviations occurred during the intervention.

**Table 1:** Demographic data of participants.

Variables	Overall	TT group	TSGDT group	t/χ2	*p*-value
Age (years)	29.73 ± 7.22	30.13 ± 7.31	29.33 ± 6.89	0.574	0.567
**Male/female**	7/97	3/49	4/48	0.475	0.491
**Bachelor degree/master's degree**	94/10	46/6	48/4	0.443	0.506
**Never seen PICC** ^a^	68(65.38%)	31(81.48%)	37(72.22%)	1.529	0.216
**Seen 1 to 2 times PICC before this training** ^a^	36(34.62%)	21(18.52%)	15(27.78%)		
**Years of clinical nursing experience**	8.85 ± 2.86	8.54 ± 2.59	9.16 ± 2.61	1.002	0.309

^a^All students are considered to lack good training experience in spinal puncture techniques. Data are expressed as mean ± standard deviation or number (%).

### 3.2 Immediate mastery of PICC skills

Post-training Objective Structured Clinical Examination (OSCE) scores demonstrated significantly superior performance in the TSGDT group vs. TT group (*p* < 0.001; showed in Table 2). TSGDT participants achieved higher mean scores across all key domains: pre-procedure preparation (*p* = 0.015), procedural execution (*p* = 0.001), and post-procedure management/holistic care (*p* = 0.010). Additionally, TSGDT participants completed the OSCE scenario more rapidly (*p* = 0.018).

**Table 2:** The OSCE for PICC of all participants after training.

Variables	TT group	TSGDT group	t/95% CI	*p*-value
Pre-operative preparation	26.89 ± 2.38	28.29 ± 3.31	2.087 (0.28–2.52)	0.015^*^
**Operational phase**	39.03 ± 6.48	43.21 ± 6.72	3.306 (1.61–6.75)	0.0017^*^
**Post procedure management & holistic care**	22.67 ± 2.53	23.82 ± 1.86	2.641 (0.29–2.01)	0.010^*^
**Total score**	88.59 ± 7.73	95.32 ± 8.35	4.271 (3.60–9.86)	< 0.0001^*^
**Operational time (minutes)**	15.37 ± 2.57	14.23 ± 2.23	2.416 (−2.08–0.20)	0.018^*^

^*^Indicates a statistically significant difference between the two groups, with a p-value less than 0.05.

### 3.3 Retention of PICC skills and knowledge

At 4-week follow-up (with no additional PICC training during the interval), OSCE scores declined slightly in both groups, but the TSGDT group maintained significantly higher retained performance (*p* < 0.001; Table 3). Domain-specific retention remained superior in TSGDT for pre-procedure preparation (*p* = 0.023), procedural execution (*p* = 0.003), and post-procedure management/holistic care (*p* = 0.015). Nevertheless, there was no difference in Knowledge test scores between two groups (*p* = 0.238). Critically, the decline in performance from immediate post-test to 4-week follow-up was significantly less pronounced in TSGDT (*p* < 0.001), indicating enhanced skill consolidation.

**Table 3:** The OSCE score and knowledge test of the participants at 4 weeks after training.

Variables	4 weeks after training			Difference in OSCE score between immediate and 4 weeks after training		
	TT group	TSGDT group	*p*-value	TT group	TSGDT group	*p*-value
**Pre-operative preparation**	25.24 ± 4.18	27.32 ± 4.97	0.023^*^	1.65 ± 0.62	0.97 ± 0.32	< 0.0001^*^
**Operational phase**	37.18 ± 8.08	42.19 ± 8.87	0.003^*^	1.85 ± 0.73	1.02 ± 0.41	< 0.0001^*^
**Post procedure management & holistic care**	21.65 ± 9.23	23.29 ± 10.52	0.015^*^	1.02 ± 0.57	0.53 ± 0.14	< 0.0001^*^
**Total score**	84.07 ± 6.93	92.80 ± 7.75	< 0.0001^*^	4.52 ± 1.79	2.52 ± 1.12	< 0.0001^*^
**Operational time (minutes)**	17.52 ± 3.18	15.78 ± 2.71	0.003^*^	2.15 ± 1.14	1.55 ± 0.98	0.005^*^
**Knowledge test**	94.59 ± 6.93	96.32 ± 7.89	0.238^*^			

^*^Indicates a statistically significant difference between the two groups, with a p-value less than 0.05.

### 3.4 Participants' subjective effects and satisfaction evaluation

TSGDT participants reported significantly higher self-efficacy, confidence, and satisfaction (Table 4). Specifically, they demonstrated greater perceived mastery (*p* = 0.026), willingness to perform PICC under supervision (*p* = 0.030), confidence in PICC provision (*p* = 0.038), and overall training satisfaction (*p* = 0.027) compared to TT participants.

**Table 4:** Participants' subjective effects and satisfaction after training.

Variables	TT group	TSGDT group	t/χ2	*p*-value
**Mastery level, yes/no**	42/10	49/3	4.308	0.038^*^
**willingness to provide PICC, yes/no**	40/12	48/4	4.727	0.030^*^
**Confidence in provide PICC, yes/no**	43/9	50/2	4.981	0.026^*^
**Satisfaction, Satisfied/average/dissatisfied**	45/7/0	51/1/0	4.875	0.027^*^

^*^Indicates a statistically significant difference between the two groups, with a p-value less than 0.05.

## 4 Discussion

This randomized controlled trial provides robust evidence that Time-Segmented Goal-Directed Teaching (TSGDT) significantly outperforms Traditional Teaching (TT) in PICC insertion training for specialist nurse trainees, with implications extending beyond immediate skill acquisition to durable learning and clinical confidence. The superior outcomes across immediate mastery, 4-week retention, and subjective confidence metrics collectively position TSGDT as a transformative pedagogical model for high-stakes clinical skill acquisition.

### 4.1 Mechanisms underlying enhanced learning

The TSGDT group's significantly higher OSCE scores (*p* < 0.001) and shorter completion time (*p* = 0.018) reflect a fundamental shift in learning architecture. By segmenting PICC insertion into discrete, time-bound phases with explicit mastery criteria, TSGDT directly addresses cognitive load theory (13, 14). This prevents the “overload” inherent in TT's holistic demonstration, allowing trainees to fully assimilate each procedural component before integration. Critically, the method's embedded deliberate practice cycle—combining targeted skill rehearsal, real-time feedback, and reflective synthesis—creates a self-correcting learning loop. This aligns with mastery learning principles (15, 16), where learning is held constant (trainees master each segment) while time is the variable (allowing individual pacing). The 4-week retention advantage (*p* < 0.001) further validates this: TSGDT's phase-by-phase mastery requirement builds a consolidated procedural schema, reducing skill decay. In contrast, TT's “one-size-fits-all” approach leaves foundational gaps that manifest as steeper retention slopes.

### 4.2 The self-efficacy nexus

The TSGDT group's markedly higher confidence (*p* = 0.038), willingness to perform (*p* = 0.030), and perceived mastery (*p* = 0.026) are not merely correlates but causal drivers of clinical readiness. Self-efficacy theory is directly instantiated here: successive achievement of segmented goals provided enactive mastery—tangible evidence of capability—thereby strengthening trainees' belief in their PICC performance efficacy (17, 18). This is clinically critical: studies confirm that ≥70% of nurses' clinical confidence directly predicts successful skill transfer to real-world settings (19). The TSGDT framework's structured feedback loop thus bridges simulation competence and clinical self-efficacy, a gap often overlooked in traditional curricula (20).

### 4.3 Clinical and educational implications

These findings transcend methodological comparison to address a pressing nursing education imperative. TSGDT's ability to simultaneously enhance skill quality, retention duration, and learner confidence offers a scalable solution for high-stakes procedures like PICC, where suboptimal performance correlates with 15–30% of catheter-related complications (21). By accelerating the transition from simulation proficiency to clinical readiness, TSGDT could directly reduce:

- First-attempt insertion failure rates
- Patient discomfort from repeated attempts
- Infection risk from prolonged procedural time

This positions TSGDT not merely as a teaching method but as a safety intervention for vascular access care.

### 4.4 Limitations and future directions

Our single-center design limits generalizability, though the cohort's homogeneity (specialist trainees with uniform baseline experience) strengthens internal validity. Critically, the 4-week retention window, while significant, cannot confirm long-term durability—a limitation we address through our proposed 12-month follow-up in ongoing research. The simulation-based assessment (OSCE) also necessitates validation against actual clinical outcomes. Future work must therefore:

- Validate in multi-center trials
- Measure clinical translation via prospective studies tracking insertion success rates, complication incidence, and patient-reported outcomes
- Expand to other high-risk skills to assess framework generalizability

## 5 Conclusion

TSGDT transcends incremental improvement to establish a new paradigm for procedural skill training. Its evidence-based integration of cognitive load reduction, mastery learning, and self-efficacy theory delivers not just better scores, but better clinicians. We urge nursing education programs to adopt this structured, goal-oriented framework to accelerate the development of competent, confident specialists capable of delivering safer, more efficient vascular access care. The data presented here provide the foundation for redefining how complex clinical skills are taught in simulation-based curricula.

## Data Availability

The raw data supporting the conclusions of this article will be made available by the authors, without undue reservation.
