# Supplementary material for: Comparison of time-segmented goal-directed teaching vs. traditional teaching for PICC among specialist nurse training: a randomized controlled trial
Source: Front Med (Lausanne). 2026 Apr 13;13:1798774. doi: 10.3389/fmed.2026.1798774 (PMC13111136; doi:10.3389/fmed.2026.1798774)
Supplement: Supplementary file 1 [file Data_Sheet_1.pdf]

Assessment of Clinical Practice Ability for Resident Physicians Training at the Third Xiangya Hospital of Central South University

Objective Structured Clinical Examination (OSCE) for PICC				
Candidate's Name			Admission Ticket Number	
Section		Detailed Criteria & Scoring Standards	Full marks	Score
Pre-operative preparation (30)	Environment and self-preparation (5)	Ensures a clean, quiet environment suitable for aseptic technique.	2	
		Performs hand hygiene (7-step technique), dons mask and cap.	2	
		Self-assessment: Confirms no personal contraindications (e.g., URTI).	1	
	Patient verification and assessment (10)	Dual verification of physician's order and informed consent (simulated).	3	
		Patient Identification: Uses at least two identifiers (name, hospital ID).	2	
		Comprehensive Assessment: a. Medical condition, treatment duration, vesicant/irritant drug therapy. b. Vascular Assessment: Prefers basilic vein; assesses diameter, elasticity, course, skin condition. c. Contraindication Screening: Inquires about history of mastectomy, upper limb DVT, limb dysfunction, pacemaker, etc.	5	
	Items preparation (5)	Verbally lists all required items: PICC insertion kit, measuring tape, sterile gloves, antiseptic, NS, heparin flush, 10ml syringe, transparent dressing, elastic bandage, etc.	3	
		Checks expiration dates and package integrity of all items.	2	
	Patient Education & Communication (6)	Explains Purpose: Clearly explains the purpose, benefits, procedure, and cooperation needed in lay terms.	2	
		Informs Risks: Briefly explains potential risks (bleeding, infection, thrombosis) and preventive measures.	2	
		Psychological Support: Assesses patient anxiety, provides reassurance and encouragement to gain cooperation.	2	
	Patient Positioning & Measurement (4)	Position: Assists patient to supine position, with arm abducted at 90°.	1	
		Accurate Measurement: a. From intended insertion site to right sternoclavicular joint. b. Down to the third intercostal space (inferior border of clavicular head). c. Arm circumference measurement (10cm above antecubital fossa).	3	
The operational phase (45)	Disinfection and draping (19)	Aseptic principles: Properly put on the sterile surgical gown and wear the sterile gloves; the laying of sterile towels.	10	
		Disinfection scope, disinfection method, disinfectant selection and drying conditions.	9	
	Puncture catheterization & Trimming (18)	Puncture: Inserts needle at correct angle (15-30°). After flashback, lowers angle and advances slightly.	5	
		Sheath Advancement: Releases tourniquet, advances introducer sheath gently.	3	
		Advancement: Advances catheter slowly and steadily. Instructs patient to turn head toward insertion side, chin to shoulder. Withdraws stylet after reaching predetermined length.	7	
		Trimming: Leaves minimum 5cm external catheter. Trims perpendicularly (no bevel, no fraying).	3	
	Securement and Verification (8)	Attaches connector. Aspirates for blood return, flushes with NS using pulsatile technique, positive-pressure locks.	4	
		Securement: Uses StatLock or sterile tape to secure connector. Applies transparent dressing using non-tension technique. Labels dressing with insertion data.	3	
		Initial Education: Informs patient about post-insertion pressure time and immediate precautions.	1	
Post-Procedure Management & Holistic Care (25)	Immediate Post-Insertion Care & Documentation (9)	Assists patient with clothing, positions for comfort.	1	
		Assessment: Checks site for bleeding, hematoma; assesses patient comfort.	2	
		Documentation: Verbally states required documentation (date/time, catheter type/length, arm circumference, procedure details, chest X-ray result).	3	
		Emphasizes: Catheter tip position must be confirmed by X-ray (distal 1/3 of SVC or CAJ) prior to any use.	3	
	Patient Education (10)	Activities of Daily Living (ADLs): Instructs on permitted ADLs (eating, writing). Avoid lifting heavy objects, excessive abduction/rotation, elbow flexion.	3	

		**Hygiene:** Recommends use of waterproof cover to keep dressing dry.	2	
		**Monitoring Signs:** Teaches patient to recognize/report redness, pain, discharge, fever, catheter migration/dislodgement, blood in tubing, and reporting pathway.	3	
		**Dressing Care:** Informs about need for scheduled professional maintenance (typically every 7 days).	2	
	**Integrated Communication & Holistic Care (6)**	**Communication Throughout:** Communicates with patient at all stages, explains steps, inquire about comfort.	3	
		**Privacy:** Maintains patient privacy during procedure.	1	
		**Professional Demeanor:** Uses respectful address, gentle tone, and demonstrates caring attitude consistently.	2	
Total score			100	
