# Supplementary material for: Comparison of time-segmented goal-directed teaching vs. traditional teaching for PICC among specialist nurse training: a randomized controlled trial
Source: Front Med (Lausanne). 2026 Apr 13;13:1798774. doi: 10.3389/fmed.2026.1798774 (PMC13111136; doi:10.3389/fmed.2026.1798774)
Supplement: Supplementary file 2 [file Data_Sheet_2.pdf]

Post class questionnaire survey on sleep health

1. What is your mastery level of PICC through this course training?? ( )

A. skilled B. average C.unskilled

2. If a patient needs to undergo PICC, would you be willing to provide it under the guidance of a teacher? ( )

A. yes B. no

3. If a patient needs to undergo PICC now, do you have confidence in successfully completing the procedure? ( )

A. yes B. no

4. How satisfied are you with this course? ( )

A. satisfied B. average C. dissatisfied
